# Orforglipron for the treatment of moderate-to-severe obstructive sleep apnea in adults with obesity or overweight: Study design and baseline characteristics of ATTAIN-OSA, a phase 3 trial

**DOI:** 10.1016/j.conctc.2026.101660

**Published:** 2026-06-19

**Authors:** Atul Malhotra, Daniel J. Gottlieb, Vaishnavi Kundel, Holger Woehrle, Sujatro Chakladar, Livia Firmino Goncalves, Giulia Calamai

**Affiliations:** a University of California San Diego, La Jolla, CA, USA; b Brigham and Women's Hospital and Harvard Medical School, Boston, MA, USA; c Icahn School of Medicine at Mount Sinai, New York, NY, USA; d Lung Center Ulm, Ulm, Germany; e Eli Lilly and Company, Indianapolis, IN, USA

**Keywords:** Obstructive sleep apnea, Weight loss, Overweight and obesity, Incretin, Cardiovascular disease risk factor

## Abstract

**Introduction:**

Obstructive sleep apnea (OSA) is highly prevalent, yet current treatment remains limited. Poor adherence to positive airway pressure (PAP) and barriers associated with injectable therapies can limit potential therapeutic options for moderate-to-severe OSA. The SURMOUNT-OSA trials demonstrated that tirzepatide contributes to OSA severity improvements; however, the injectable mode of administration introduces barriers that may limit accessibility and long-term adherence. Orforglipron, a once daily oral glucagon-like-peptide-1 receptor agonist, may offer a more feasible and accepted therapeutic option. ATTAIN-OSA was developed to evaluate the efficacy and safety of oral orforglipron in adults with moderate-to-severe OSA.

**Methods:**

ATTAIN-OSA is a master protocol with two multicenter, randomized, double-blind, placebo-controlled Phase 3 trials enrolling adults with moderate-to-severe OSA and obesity or overweight. Study 1 includes participants unable or unwilling to use PAP. Study 2 includes participants who use PAP and complete a protocol-mandated washout before baseline polysomnography. Participants are randomly assigned to placebo or orforglipron capsule formulation at maximum tolerated dose (12, 24, or 36 mg) for 52 weeks following a standardized dose escalation schedule.

**Results:**

The primary endpoint is change in Apnea-Hypopnea Index (AHI) at Week 52. Key secondary endpoints include sleep apnea-specific hypoxic burden, Patient-Reported Outcomes Measurement Information System sleep-related impairment, high-sensitivity C-reactive protein, and body weight, and other AHI-related endpoints. Overall, 712 participants have been randomized to orforglipron or placebo (Study 1, n = 363; Study 2, n = 349).

**Conclusion:**

ATTAIN-OSA evaluates if once-daily oral orforglipron can provide an effective and more accessible therapeutic approach to treat moderate-to-severe OSA in adults with obesity or overweight.

**Trial registration:**

ClinicalTrials.gov, NCT06649045.

## Highlights

• ATTAIN-OSA evaluates efficacy and safety of once-daily oral orforglipron for OSA.
• Adults with moderate-severe OSA and body mass index ≥27 kg/m^2^.
• Treatment duration was 52 weeks.
• Endpoints: change in AHI, patient reported outcomes, cardiometabolic risk markers.
• Importance: oral therapy may enhance adherence vs injectable weight loss treatments.

## 1 Introduction

Obstructive sleep apnea (OSA) is a common condition with major neurocognitive and cardiometabolic sequelae [1,2]. OSA is estimated to affect up to 1 billion people worldwide, with recent estimates suggesting increasing prevalence over time, particularly in women [3,4]. The obesity pandemic, together with an aging population is an important driver of OSA occurrence [5]. Despite the increasing prevalence, many individuals afflicted with OSA remain undiagnosed and untreated [6].

Currently, the first-line treatment for OSA is positive airway pressure (PAP), which can have transformative benefits for some individuals, but is poorly tolerated by others [7,8]. PAP therapy aims to provide a pneumatic splint to resolve apneic events [9]. It is a highly efficacious treatment; however, many individuals refuse PAP immediately or soon after treatment begins, are nonadherent, or discontinue early, limiting its long term effectiveness [10,11]. Consequently, a proportion of individuals with OSA are unable or unwilling to use PAP with nonadherence as the major clinical challenge [7]. Barriers to OSA therapy include limited PAP acceptance due to cost, inconvenience, discomfort, as well as limited availability of alternative therapies [7,[12], [13], [14]]. In addition, randomized trials have not shown major improvements in cardiometabolic risk with PAP therapy, leading some investigators to recommend addressing underlying causes and/or comorbid conditions including obesity [[15], [16], [17]]. Further, clinical investigations indicate that individuals with OSA and obesity or overweight should be offered a weight-loss intervention in addition to PAP therapy for adjunct improvements in cardiometabolic markers, including insulin sensitivity, serum triglyceride levels, and reductions in blood pressure (BP) [15].

The SURMOUNT-OSA study was recently completed and demonstrated that tirzepatide, a dual glucose-dependent insulinotropic peptide (GIP) and glucagon-like-peptide-1 (GLP-1) receptor agonist (RA), was superior to placebo in participants not currently using PAP therapy (Study 1) and in those on PAP therapy (Study 2) [18]. SURMOUNT-OSA demonstrated that tirzepatide significantly improved the primary endpoint (change in Apnea-Hypopnea Index [AHI]), key sleep-disordered breathing endpoints and additional health benefits, including the reduction in cardiovascular (CV) risk factors and improvement in patient-reported sleep-related functioning in participants with moderate-to-severe OSA and obesity [18]. Secondary outcomes, which were pre-specified and controlled for multiple comparisons, showed significant improvements in systolic blood pressure, high-sensitivity C-reactive protein (hsCRP), sleep apnea–specific hypoxic burden (SASHB), patient-reported outcomes (PROs), and body weight [18]. Based on these data, tirzepatide received FDA approval for the treatment of moderate-to-severe OSA in adults with obesity [19].

However, tirzepatide is administered as a subcutaneous injection. As with all injectable therapies, it has several limitations, including the need for cold chain distribution and storage (which can be challenging in under-resourced areas), risk of injection site reactions, and needle-related discomfort, fear, and stigma, that may limit treatment initiation and adherence [[20], [21], [22]]. These issues underscore the need for orally administered GLP-1 RAs to address ongoing unmet needs of people living with OSA and obesity/overweight.

Oral agents may be amenable to easier storage, distribution, and administration. They can be manufactured at a greater scale, improving access [22,23]. They may be more accessible for individuals, particularly in under resourced settings where injections present logistical challenges, and can improve adherence in populations hesitant about injections [21,23]. These agents allow individuals to select a formulation aligned with their needs [21]. Thus, there is a strong rationale to identify oral agents capable of achieving comparable outcomes to injectable therapies in OSA.

Orforglipron is a once-daily, orally administered, small-molecule, GLP-1 RA to be taken with a reduced-calorie diet and increased physical activity [24]. It is indicated to reduce excess body weight and maintain weight-reduction long-term in adults with obesity or with overweight and at least one weight-related condition [24]. It is also being investigated for type 2 diabetes, hypertension, osteoarthritis pain, stress urinary incontinence and peripheral artery disease [[25], [26], [27], [28], [29], [30], [31], [32]]. In the ATTAIN-1 study, in participants with obesity and without type 2 diabetes, orforglipron 36 mg reduced bodyweight by up to 12.4% after 72 weeks of treatment, with associated improvements in cardiometabolic risk factors [33]. Considering the importance of weight reduction for individuals with OSA and the increasing prevalence of the disease, evaluating the effects of orforglipron in individuals with moderate-to-severe OSA and obesity or overweight is of great clinical interest [3,4,[34], [35], [36], [37]].

Based on this rationale, we sought to test the hypothesis that orforglipron would be superior to placebo in participants with moderate-to-severe OSA and obesity or overweight in ATTAIN-OSA trials between two different patient populations - individuals unable or unwilling to use PAP and those currently on PAP therapy [38]. We examined the AHI as the primary endpoint for sleep apnea severity. We also evaluated secondary outcomes controlled for multiple comparisons, including SASHB, Patient-Reported Outcomes Measurement Information System (PROMIS) sleep-related impairment, body weight, and hsCRP. Here, we report the study design and baseline characteristics of participants enrolled in the ATTAIN-OSA studies.

## 2 Methods

### 2.1 Study design and participants

**Fig. 1:**
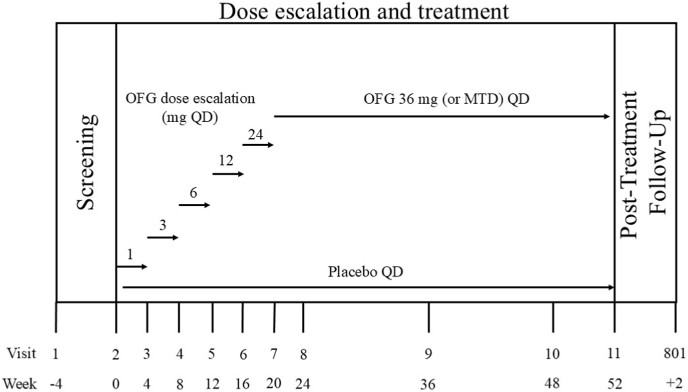
Study design Caption: Panel A displays the dose escalation and visit schema. Panel B displays the ATTAIN-OSA schema. Note: Panel A and B display doses in capsule formulation. Abbreviations: GZ01, Study JA2-MC-GZ01; GZ02, Study JA2-MC-GZ02; MTD, maximum tolerated dose; OFG, orforglipron; PAP, positive airway pressure; QD, once daily.

ATTAIN-OSA is a multicenter, randomized, parallel-arm, double-blind, placebo-controlled Phase 3 study with 52-week duration to evaluate the efficacy and safety of orforglipron at the maximum tolerated dose (MTD: 12, 24, or 36 mg capsule) compared to placebo in participants with moderate-to-severe OSA and overweight or obesity. ATTAIN-OSA is a master protocol investigating two different participant populations, described in two independent studies, GZ01 and GZ02. Study 1, GZ01, includes 363 participants who were unwilling or unable to use PAP therapy and had not used PAP for at least 4 weeks before entering the study. Study 2, GZ02, includes 349 participants using PAP therapy for at least 3 months at the time of screening and planning to continue PAP therapy during the study (Fig. 1).

This trial is registered with ClinicalTrials.gov (NCT06649045), and the estimated study completion date in 2026. Eligible participants include adults aged ≥18 years with a history of moderate-to-severe OSA, as confirmed by an AHI ≥15 on a centrally-scored polysomnography (PSG) evaluation during the screening period before starting the study treatment. They have a body mass index (BMI) ≥27 kg/m2 and a history of ≥1 self-reported unsuccessful dietary weight-loss efforts. Exclusion criteria include a history of type 1 or 2 diabetes and a self-reported change in body weight of *>*5 kg within 3 months before screening. Further trial eligibility criteria are shown in Table 1.

**Table 1:** Key eligibility criteria.

**Key inclusion criteria**
• Adult male or female, ≥18 years old • Confirmed history of moderate-to-severe OSA • AHI ≥15, confirmed by central PSG evaluation at V1 • BMI ≥27 kg/m^2^ • History of at least one self-reported unsuccessful dietary effort to lose weight
**Key exclusion criteria**
Diabetes related
• Type 1 or 2 diabetes • HbA_1c_ ≥ 6.5% • History of ketoacidosis or hyperosmolar state/coma
Sleep-disordered breathing related
• Any previous or planned upper airway surgery for sleep apnea or major ear, nose, or throat surgery • Significant craniofacial abnormalities that may affect breathing at V1 • Diagnosis of central or mixed sleep apnea, or diagnosis of Cheyne-Stokes Respiration • Diagnosis of narcolepsy, idiopathic hypersomnia, REM or non-REM parasomnia, or insomnia requiring treatment or preventing >240 min total sleep time at PSG • Diagnosis of obesity hypoventilation syndrome or daytime hypercapnia • Undergoing active treatment of OSA other than positive airway pressure therapy
Obesity related
• Self-reported or documented change in body weight >5 kg within 90 days prior to screening • Prior or planned surgical treatment for obesity • Prior or planned endoscopic and/or present device-based therapy for obesity • Obesity induced by other endocrinologic disorders • Diagnosis of monogenetic or syndromic forms of obesity
Medical
• eGFR <30 mL/min/1.73 m^2^ • Clinically significant gastric emptying abnormality or use of drugs affecting gastric motility • Family or personal history of medullary thyroid carcinoma or multiple endocrine neoplasia syndrome type 2 • Serum calcitonin level ≥35 ng/L • TSH level outside of the defined normal range at V1 or signs of hypothyroidism that may require intervention during the study period • History of active or untreated malignancy or in remission from malignancy for <5 years (basal or squamous cell skin cancer, in situ carcinomas of cervix, or in situ or Grade 1 prostate cancer allowed) • Active suicidality or deemed significant risk for suicide following completion of C-SSRS or by judgment of investigator • PHQ-9 ≥15 at V1 • Acute myocardial infarction, cerebrovascular incident (stroke), coronary artery revascularization, unstable angina, or hospitalization due to congestive heart failure within 90 days prior to V1 or between V1 and V2 • History of (<90 days prior to V1) or planned cardiovascular procedure • NYHA functional classification class IV congestive heart failure prior to V1 • Acute or chronic hepatitis or symptoms of any other liver disease other than nonalcoholic fatty liver disease • ALT or AST ≥3.0× ULN, ALP ≥1.5× ULN, or TBL ≥1.5× ULN (except for Gilbert's syndrome) for the reference range • Hepatitis B infection • Positive hepatitis C antibody and positive HCV RNA • Significant, active autoimmune abnormality that may require concurrent treatment with systemic glucocorticoids during study course by judgment of investigator • History of marijuana use <3 months of V1 and unwillingness to abstain during study duration • Respiratory or neuromuscular disease that could interfere with study results by judgment of investigator • History of chronic or acute pancreatitis • Have a transplanted organ or awaiting organ transplant (corneal transplants [keratoplasty] allowed) • Requires use of supplemental oxygen
Prior/concomitant therapy
• Use of chronic systemic glucocorticoid therapy within 90 days prior to V1 or between V1 and V2 • Use of metformin or other glucose-lowering medication, regardless of indication within 90 days prior to V1 or between V1 and V2 • Use of medications that may cause significant weight gain, such as tricyclic antidepressants, atypical antipsychotics, and mood stabilizers, within 12 months prior to V1 • Use of anti-obesity medication or alternative weight-loss remedies, within 180 days prior to V1, or between V1 and V2 • Use of implantable or injectable contraceptives within 18 months prior to V1 (IUD acceptable) • Use of strong CYP3A inhibitors or inducers, strong OATP inhibitors, or drugs that are sensitive P-gp/BCRP substrates with narrow therapeutic index o To be eligible for randomization, these drugs would require washout for at least 2 weeks prior to V2 and participant should be on stable dose of alternative medication for at least 2 weeks prior to V2 • Use of GLP-1 RA or tirzepatide <180 days prior to V1 • Use of stimulants <90 days prior to V1 • Use of hypnotics, mirtazapine, opioids, trazodone/vilazodone <90 days prior to V1

Abbreviations: AHI, Apnea-Hypopnea Index; ALP, alkaline phosphatase; ALT, alanine aminotransferase; AST, aspartate aminotransferase; BCRP, breast cancer resistance protein; BMI, body mass index; C-SSRS, Columbia-Suicide Severity Rating Scale; CYP3A, cytochrome P450, family 3, subfamily A; eGFR, estimated glomerular filtration rate; GLP-1 RA, glucagon-like peptide 1 receptor agonist; HbA1c, glycated hemoglobin; HCV, hepatitis C virus; IUD, intrauterine device; NYHA, New York Heart Association; OATP, organic-anion-transporting polypeptides; OSA, obstructive sleep apnea; P-gp, P-glycoprotein; PHQ-9, Patient Health Questionnaire-9; PSG, polysomnography; REM, rapid eye movement; RNA, ribonucleic acid; TBL, total bilirubin level; TSH, thyroid-stimulating hormone; ULN, upper limit of normal; V1, Visit 1; V2, Visit 2.

As OSA is more common in males, the trial caps male enrollment at approximately 70% to ensure sufficient female participation [3,4,34,39]. Participant sex is defined in the study protocol as individuals assigned female at birth (AFAB) or individuals assigned male at birth (AMAB).

The trial is being conducted in accordance with good clinical practice guidelines and the principles of the Declaration of Helsinki. Independent ethics committee or institutional review board approvals have been received for each participating site. The initial ethics review board approval occurred on September 09, 2024, and the most recent approval occurring on February 04, 2026. All participants provide written informed consent before trial participation, and their privacy rights are respected.

### 2.2 Procedures

ATTAIN-OSA is evaluating orforglipron at the MTD (12, 24 or 36 mg capsule). Orforglipron or matching placebo is administered daily as an oral capsule. Placebo capsules matched study intervention capsules to maintain blinding. Study investigators, site personnel, clinical monitors, and participants were blinded to the study intervention until study completion. If unblinding occurred, participants were permanently discontinued from study intervention but continued to be evaluated for efficacy and safety for the study duration.

This study will present data for the maximum tolerated dose of orforglipron from the investigational orforglipron capsule formulations. The investigational orforglipron capsule formulations are 1 mg, 3 mg, 6 mg, 12 mg, 24 mg, and 36 mg. These doses have been shown as the equivalent to tablet doses of 0.8 mg, 2.5 mg, 5.5 mg, 9 mg, 14.5 mg, and 17.2 mg, which are approved in the USA [24,40]. The starting dose of orforglipron is 1 mg capsule for 4 weeks, followed by escalation to 3 mg capsule. Subsequent dose increases occur at 4-week intervals with no more than a doubling of dose until the MTD is reached (12, 24, or 36 mg capsule) (Fig. 1). This dose regimen permits adequate time to tolerate any GI events and is expected to improve GI tolerability in the Phase 3 studies. Participants are allowed to continue in the trial at MTDs <36 mg once daily (QD) (capsule dose) if this dose is at least 12 mg (capsule dose). Throughout the trial, all participants, regardless of assigned study treatment, receive individualized lifestyle counseling focused on a healthy, balanced diet combined with physical activity. Participants were encouraged to complete a 3-day diet and physical activity log prior to each study visit. At each visit, adherence to the lifestyle management, diet and physical activity were assessed with additional counseling provided as needed, with appropriate documentation in the participant's record.

Compliance to the study interventions is defined as adhering to at least 75% of the study intervention during the treatment period. For PAP adherence, compliance was collected based on participants’ PAP adherence diary, where participants recorded values from the PAP device output. This was monitored by investigators at scheduled visits where investigators considered how many days per week and hours per night the device was used.

The ATTAIN-OSA master protocol includes a 4-week screening period followed by a 52-week treatment period and 2-week off-drug safety follow-up period (Fig. 1). Dose de-escalation and re-escalation are allowed during the first 20 weeks after randomization, providing participants with a greater opportunity to achieve the MTD. De-escalation and subsequent re-escalation are allowed only for the management of gastrointestinal symptoms when other mitigations such as dietary counseling, symptomatic treatment, or temporary drug interruption have failed. The study treatment is discontinued for participants unable to tolerate the MTDs. Dose modifications beyond 20 weeks after randomization are not permitted. Treatment discontinuation is decided by the participant or the investigator, based on the criteria outlined in the protocol. Clinical considerations for treatment discontinuation include initiation of prohibited medication, gastrointestinal symptoms not tolerated by the participant, significantly elevated calcitonin levels, diagnosis of type 1 diabetes, pancreatitis, malignancy, major psychiatric disorder at mental health professionals’ further evaluation, or an incident pregnancy. Participants who permanently discontinue the study drug during the double-blind treatment period are encouraged to continue attending all scheduled study visits for the collection of all planned measurements.

A steering committee comprised an academic/industry partnership with the lead author serving as chairperson with participation and other members participating, including co-authors. The committee scheduled meetings throughout study design and execution to make decisions regarding study conduct and to troubleshoot issues as they arose. The steering committee remains blinded to all data, with an independent data monitoring committee (DMC) in place to review unblinded data. The DMC is reviewing and evaluating unblinded trial data on a 6-month and an ad hoc basis. The aim is to protect the study participants’ safety, evaluate the benefit-risk balance, and provide recommendations on trial continuation, modification, or termination.

### 2.3 Objectives and endpoints

The primary objective of ATTAIN-OSA is to demonstrate the superiority of orforglipron at the MTD versus placebo after 52 weeks, as an adjunct to diet and exercise, in reducing AHI in participants with moderate-to-severe OSA and obesity or overweight. AHI is measured by PSG at baseline and at Weeks 24 and 52. Participants in Study GZ02 are required to withdraw from PAP therapy for 7 days prior to each PSG and related procedures at the 3 timepoints during the 52-week treatment period. Short-term PAP withdrawal results in OSA recurrence and allows for the investigation of potential treatment effects without unduly jeopardising safety in individuals who tolerate PAP [41]. Based on the literature, a 7-day withdrawal is appropriate to address safety and tolerability considerations, and provides a reasonable approach to assess the effect of orforglipron while minimising the potential confounding effects of PAP [41]. The experimental model, which was also used in SURMOUNT-OSA, allows for the evaluation of orforglipron treatment effect in a diverse and generalizable population of participants with moderate-to-severe OSA and obesity or overweight, including those undergoing PAP therapy.

PSG assessments (including AHI, blood oxygen saturation parameters, pulse rate [PR], sleep parameters) are performed during one, overnight clinic stay, per the Schedule of Activities. Data from PSGs are read and scored centrally using the American Academy of Sleep Medicine 1B hypopnea scoring method (using ≥4% oxygen desaturation from pre-event baseline) [42]. Hypopneas are scored if the airflow drops by ≥ 30% from baseline for ≥10 s, associated with a ≥4% oxygen desaturation [43]. Apneas are defined as cessations in breathing lasting ≥10 s [44]. Moderate OSA is defined as an AHI ≥15 and < 30 respiratory events per hour, and severe OSA as an AHI ≥30 events per hour [34]. The eligibility criteria and AHI-related endpoints of the study are assessed based on central reading of the PSG.

Key secondary objectives, controlled for type 1 error, include percent change in AHI, percent change in SASHB (% min/hour), change in PROMIS Short Form Sleep-Related Impairment 8a T-score, percent of participants achieving ≥50% AHI reduction or achieving OSA remission (AHI <5) or mild nonsymptomatic OSA (AHI 5-14 with Epworth Sleepiness Scale [ESS] ≤10), percent change in body weight and inflammatory status. Other secondary objectives include change in ESS score, PROMIS Short Form Sleep Disturbance 8b T-score, SBP, and lipid parameters (Table 2). For the primary endpoint, prespecified subgroup analyses will be performed for age (<50 and ≥ 50 years; <65 and ≥ 65 years), sex (AFAB, AMAB), baseline OSA severity (severe, not severe), region (US, Outside US), baseline BMI, race, ethnicity, and baseline ESS. Baseline BMI will be evaluated using three subgroup analyses: <30 kg/m^2^ or ≥30 kg/m^2^, <35 kg/m^2^ or ≥35 kg/m^2^, and <40 kg/m^2^ or ≥40 kg/m^2^. Baseline ESS was categorized as ≤10 and > 10, as sleepiness was defined as an ESS >10.

**Table 2:** Objectives and endpoints.

**Primary Objective**	**Endpoints**

To demonstrate that orforglipron MTD QD is superior to placebo for change in AHI	From baseline to week 52: • Change in AHI (events per hour)

**Key secondary objectives**	**Endpoints**

To demonstrate that orforglipron MTD QD is superior to placebo for:	From baseline to week 52:
• Change in AHI	• Percent change in AHI
• Change in hypoxic burden	• Percent change from baseline in SASHB (% min/hour)
• Assessment of patient-reported sleep-related functioning	• Change in PROMIS Short Form SRI 8a T-score
• Clinically meaningful change in AHI	• Percent of participants achieving ≥50% AHI reduction
• Achieving OSA remission or mild nonsymptomatic OSA	• Percent of participants with o AHI <5, or o AHI 5-14 with ESS ≤10
• Change in body weight	• Percent change in body weight
• Change in inflammatory status	• Percent change in hsCRP concentration

**Additional secondary endpoints**	**Endpoints**

To demonstrate that orforglipron MTD QD is superior to placebo for:	From baseline to Week 52:
• Change in excessive daytime sleepiness	• Change in ESS score
• Change in lipid parameters	• Percent change in o HDL-cholesterol o Non-HDL cholesterol o Triglycerides
• Assessment of patient-reported sleep-related functioning	• Change in PROMIS Short Form SRI 8a T-score with baseline PROMIS SRI ≥55 • Proportion of participants achieving clinically meaningful within-patient change in: PROMIS SRI
• Assessment of patient-reported sleep disturbance	• Change in PROMIS Short Form Sleep Disturbance 8b T-score
• Change in patient-reported health status	• Change in SF-36v2 acute form domain and summary scores
• Patient-reported global impression of severity- sleep-related outcomes	• Categorical shifts in: o PGIS-OSA Sleepiness o PGIS-OSA Fatigue o PGIS-OSA Snoring o PGIS-OSA Sleep quality
• Insulin	• Change in fasting insulin
• Health-related quality of life	• Change in: o EQ-5D-5L utility index o EQ-VAS scores
• Patient-reported global impression of change- sleep-related outcomes	• Categorical shift in: o PGIC-OSA Sleepiness o PGIC-OSA Fatigue o PGIC-OSA Snoring o PGIC-OSA Sleep quality
• Change in SBP	From baseline to Week 48: • Change in SBP^a^
• Change in DBP	From baseline to Week 48: • Change in DBP^a^
• To describe the safety of orforglipron MTD QD in participants with OSA	• Summary of safety data, including number and incidence of: o SAEs o TEAEs o Discontinuation due to AEs

Abbreviations: AE, adverse event; AHI, Apnea-Hypopnea Index; DBP, diastolic blood pressure; ESS, Epworth Sleepiness Scale; EQ-5D-5L, EuroQol 5-Dimensions, 5-Level version; EQ-VAS, EuroQol Visual Analog Scale; hsCRP, high-sensitivity C-reactive protein; HDL, high-density lipoprotein; MTD, maximum tolerated dose (12, 24, or 36 mg); OSA, obstructive sleep apnea; PGIC-OSA, Patient Global Impression of Change-Obstructive Sleep Apnea; PGIS-OSA, Patient Global Impression of Severity-Obstructive Sleep Apnea; PROMIS, Patient-Reported Outcomes Measurement System; QD, once daily; SAE, serious adverse event; SASHB, sleep apnea-specific hypoxic burden; SBP, systolic blood pressure; SF-36v2, Short-Form 36 version 2; SRI: sleep-related impairment; TEAE, treatment-emergent adverse event.

a Blood pressure was assessed at Week 48 since positive air pressure suspension at Week 52 may confound blood pressure assessment.

The PROMIS Sleep Disturbance and Sleep-Related Impairment measures were selected as the most appropriate PROs for this study. These assessments capture complementary dimensions of patient reported sleep health, distinguishing nocturnal sleep experiences from their daytime consequences. Specifically, PROMIS Sleep Disturbance assesses perceived sleep quality, depth, restfulness, and difficulties with sleep initiation and maintenance, while PROMIS sleep-related impairment evaluates the impact of sleep problems on daytime functioning, including sleepiness, fatigue, and impairments in alertness, cognition, and daily activities. The assessments have been collected at screening, Week 24, and 52. For participants in Study GZ02, PROs have been collected after the 7-day washout period, on the same day of the PSG test. All adverse events regardless of severity are reportable by the investigators, including those leading to treatment discontinuation, those requiring independent adjudication, and those requiring additional data collection. All adverse events and adjudication outcomes are reviewed by an independent DMC, consisting of professionals with relevant expertise in ATTAIN-OSA.

#### 2.3.1 Notable assessment

*PSG measures of interest.* SASHB is determined by measuring the respiratory event-associated area under the curve for oxygen desaturation from pre-event baseline [45]. As such, this metric represents the cumulative burden of intermittent hypoxia caused by OSA-related sleep-disordered breathing. SASHB strongly predicts OSA-related CV disease mortality, overall mortality, and incident heart failure after accounting for important risk factors and comorbidities [[45], [46], [47]]. A recent post hoc analysis of the ISAACC randomized controlled trial demonstrated that treating individuals with a high hypoxic burden was associated with reduced risk of long-term CV disease [48]. Given its role as a predictor of OSA-related major adverse CV outcomes, SASHB will be assessed from PSG readings in ATTAIN-OSA. The study will assess orforglipron–related improvement in hypoxic burden and analyze the correlation between changes in SASHB with those in sleep, breathing, cardiometabolic indicators, and PROs. Polysomnographic estimates of sleep apnea endotypic characteristics, such as pharyngeal collapsibility, muscle compensation, loop gain, and arousal threshold, will be calculated from PSG data [[49], [50], [51]].

### 2.4 Statistical considerations

Within each study under the master protocol, participants are randomized in 1:1 ratio to either orforglipron MTD or placebo. Randomization of participants has been based on the stratification factors of country/geographic region, baseline AHI (moderate or severe), and sex (individual AFAB or individual AMAB).

For Studies GZ01 and GZ02, the statistical power is evaluated for the primary efficacy endpoint and a key secondary endpoint (PROMIS Short Form Sleep-Related Impairment 8a T-score) at a 2-sided significance level of 0.05 using a 2-sample *t*-test. This sample size provides:

• at least 95% power to demonstrate the superiority of orforglipron MTD to placebo for the mean change from baseline in AHI, assuming 15 events/hour improvement, with a common SD of 25 events/hour, and
• approximately 85% power to demonstrate the superiority of orforglipron MTD to placebo for the mean change from baseline in PROMIS Short Form Sleep-Related Impairment 8a T score, assuming a 3.5-point improvement, with a common SD of 10 points.

Two estimands will be defined for the primary and key secondary efficacy analyses, the treatment regimen estimand and the efficacy estimand. For the primary endpoint, both estimands use the mean change in AHI from baseline to 52 weeks of treatment in the target patient population as defined by the study eligibility criteria in Table 1. The treatment regimen estimand represents the treatment difference between orforglipron and placebo regardless of adherence to assigned study treatment. This estimand allows for potential dose interruptions and modifications, assuming no treatment benefit after initiation of certain prohibited medications. The efficacy estimand represents the treatment difference between orforglipron and placebo in participants who adhere to their assigned study treatment. This estimand allows for potential dose interruptions and dose modifications, provided that participants do not initiate certain prohibited medications and do not have a consecutive interruption of 14 or more days.

For the treatment regimen estimand, a no-treatment hypothetical strategy, assuming the absence of therapeutic effect, will be used to handle intercurrent events (ICEs) of initiation of PAP therapy for non-PAP users, initiation of prohibited OSA surgery/procedure, or initiation of certain prohibited weight management treatment options, which can refer to medications and procedures (i.e. bariatric surgery/weight loss procedures). For all other ICEs in the treatment regimen estimand, including but not limited to, permanent discontinuation of study intervention, a treatment policy strategy will be implemented. Therefore, all available data collected after the occurrence of the ICE will be included in the statistical analyses.

For the efficacy estimand, ICEs will be handled using the hypothetical strategy, including permanent discontinuation of study treatment, initiation of PAP therapy for non-PAP users, initiation of prohibited OSA surgeries or procedures, initiation of certain prohibited weight management treatment options, which can refer to medications or procedures (i.e. bariatric surgery/weight loss procedures), and treatment interruptions of 14 or more consecutive days. The potential outcome of interest is the change in AHI if participants would remain on their assigned treatment for 52 weeks without any of these ICEs. Dose modification and treatment interruptions of fewer than 14 consecutive days will not be considered ICEs since these are part of the treatment condition.

Missing data will be handled using multiple imputation appropriately classifying the missing data based on their missingness pattern, including missing at random (MAR) or missing not at random (MNAR). After imputation, the primary efficacy comparison will be performed using the ANCOVA model for the mean change in AHI from baseline to Week 52. The ANCOVA model will include treatment arm and stratification factors as fixed effects, with baseline AHI as a fixed covariate. Statistical inference across multiple imputed datasets will follow Rubin's publication [52].

Multiplicity adjusted analyses will be applied to the primary and key secondary objectives using the graphical multiple testing procedure [53,54]. The primary and key secondary endpoints will be controlled for family-wise type 1 error rate at a two-sided significance level of 0.05 within each study, independently. Analysis for the key secondary endpoint of change in PROMIS Short Form Sleep-Related Impairment 8a will be tested with a submission-wise error rate (SWER) control strategy, by conducting a pooled analysis across the two studies [55,56].

## 3 Demographics and baseline characteristics

As illustrated in Table 3, the enrolled population reflects the demographic and anthropometric profile typical of individuals with OSA in standard clinical practice, comprising 29.2% females with a mean age of 49.7 (±11.8) years and a mean BMI of 38.0 (±6.9) kg/m^2^. The study population is racially and ethnically diverse, including 17.5% Asian, 4.0% Black, and 8.5% American Indian or Alaska Native, with 43.0% of Hispanic/Latino ethnicity. The mean AHI is 54.1 (±29.3) events/hour - 50.7 (±28.6) events/hour in Study GZ01 and 57.6 (±29.6) events/hour in Study GZ02 and is consistent with most participants (71.8%) having a diagnosis of severe OSA - 67.8% in Study GZ01 and 75.9% in Study GZ02. Based on the ESS total score ranging from 0 to 24, with higher scores indicating greater daytime sleepiness and a score of >10, a commonly used threshold, indicating excessive sleepiness, the mean ESS score of 11.1 (5.1) indicates that participants were, on average, mildly sleepy at baseline [57]. PROs are consistent with the severity of the disease, with a mean PROMIS Short Form Sleep-Related Impairment 8a value of 57.0 (±8.6) and a mean PROMIS Short Form Sleep Disturbance 8b value of 56.7 (±7.8).

**Table 3:** Demographics and baseline demographics.

Parameter	GZ01 (N = 363)	GZ02 (N = 349)	Overall (N = 712)
Age, years	48.4 (11.8)	51.1 (11.6)	49.7 (11.8)
<50	185 (51)	151 (43.3)	336 (47.2)
≥50	178 (49)	198 (56.7)	376 (52.8)
Female	114 (31.4)	94 (26.9)	208 (29.2)
Race			

American Indian/Alaska native	33 (9.2)	26 (7.6)	59 (8.5)
Asian	76 (21.3)	46 (13.5)	122 (17.5)
Black/African American	13 (3.6)	15 (4.4)	28 (4)
Native Hawaiian or Other Pacific Islander	0	1 (0.3)	1 (0.1)
White	231 (64.7)	248 (72.9)	479 (68.7)
Multiple	4 (1.1)	4 (1.2)	8 (1.1)
Missing	6	9	15

Ethnicity			

Hispanic or Latino	163 (44.9)	143 (41)	306 (43.0)
Not Hispanic of Latino	188 (51.8)	200 (57.3)	388 (54.5)
Not reported	12 (3.3)	6 (1.7)	18 (2.5)

Region			

United States	81 (22.3)	88 (25.2)	169 (23.7)
Outside of United States	282 (77.7)	261 (74.8)	543 (76.3)

Weight, kg	110.2 (23.07)	114.6 (23.01)	112.4 (23.13)
BMI, kg/m^2^	37.4 (6.75)	38.6 (6.92)	38 (6.86)

<35	156 (43)	115 (33)	271 (38.1)
≥35 to <40	98 (27)	105 (30.1)	203 (28.5)
≥40	109 (30)	129 (37)	238 (33.4)

HbA1c, %	5.67 (0.35)	5.71 (0.36)	5.69 (0.35)
AHI, events/hour	50.71 (28.6)	57.63 (29.57)	54.1 (29.26)
OSA severity			
Moderate	117 (32.2)	84 (24.1)	201 (28.2)
Severe	246 (67.8)	265 (75.9)	511 (71.8)
Waist circumference, cm	117.4 (15.2)	120.3 (15.28)	118.8 (15.3)
Neck circumference, cm	43.7 (6.78)	44 (4.64)	43.9 (5.83)
ESS score	11.4 (5.24)	10.9 (4.95)	11.1 (5.11)
≤ 10	169 (46.8)	154 (50.2)	323 (48.4)
> 10	192 (53.2)	153 (49.8)	345 (51.6)
PROMIS sleep disturbance	55.4 (7.99)	58.1 (7.37)	56.7 (7.83)
PROMIS SRI	56.2 (8.67)	57.9 (8.34)	57.0 (8.56)
Sleep efficiency, %	75.9 (11.95)	75.0 (11.41)	75.4 (11.69)
Sleep onset, min	19.0 (23.97)	18.7 (23.43)	18.9 (23.69)
WASO, min	95.4 (53.86)	99.8 (50.43)	97.6 (52.22)
REM sleep, %	14.1 (6.91)	11.7 (6.66)	12.9 (6.88)

Note: Data presented as mean (standard deviation) or n (%) unless otherwise noted.

Abbreviations: AHI, Apnea-Hypopnea Index; BMI, body mass index; ESS, Epworth Sleepiness Scale; GZ01, Study JA2-MC-GZ01; GZ02, Study JA2-MC-GZ02; HbA1c, glycated hemoglobin; OSA, obstructive sleep apnea; PROMIS, Patient-Reported Outcomes Measurement Information System; REM, rapid eye movement; SRI, sleep-related impairment; WASO, wakefulness after sleep onset.

## 4 Discussion

We believe ATTAIN-OSA represents an important potential advancement in the evolving landscape of OSA therapeutics. This program will help determine the impact of orforglipron on OSA severity and associated cardiometabolic risk and may corroborate and extend observations from the SURMOUNT-OSA trials. The oral mode of administration may enhance accessibility for individuals with OSA who face barriers to injectable treatments or who prefer a noninjectable option.

A notable limitation of SURMOUNT-OSA was its BMI inclusion threshold of ≥30 kg/m^2^. In contrast, ATTAIN-OSA incorporates a lower threshold of ≥27 kg/m^2^, thereby broadening eligibility to a larger and more representative proportion of individuals with OSA. Although many individuals with OSA have obesity, there is increasing recognition of the substantial subset that has overweight or only modest obesity. The expanded BMI range in ATTAIN-OSA will therefore help clarify the potential benefits of weight loss therapy across a more diverse and broader OSA population.

Despite this study's strengths, there are limitations to consider. First, ATTAIN-OSA does not address the long-term sustainability of treatment effects. Longer-term evaluations would be valuable, especially in light of the three-year SURMOUNT-1 follow-up results, which showed durable benefits of tirzepatide, including a marked reduction in diabetes incidence [58]. Second, the study is not powered to assess major CV outcomes such as myocardial infarction or stroke. While we will evaluate OSA-related CV risk factors including SBP, SASHB, and hsCRP, dedicated outcome trials will be needed to determine the impact on major CV events. Finally, ATTAIN-OSA excludes individuals with diabetes, mild OSA, or BMI <27 kg/m^2^, limiting its generalizability to patients with OSA who fall outside of our study eligibility criteria. Nonetheless, given the prevalence of moderate-to-severe OSA among adults with overweight or obesity, the population studied remains broadly representative and clinically relevant.

While study investigators follow a strict protocol for blinding, a potential limitation could be the risk of functional unblinding when considering weight reduction associated with orforglipron-assigned participants, mitigated somewhat by the individualized diet and exercise counseling provided to all participants. While all efforts to maintain blinding have been made, the potential for functional unblinding could present bias to participants, particularly for PROs. However, functional unblinding is an inherent limitation of many clinical trials involving agents with discernible physiologic effects and will be considered by the investigators for future analyses.

PAP therapy adherence was monitored closely at visits among participants on PAP at baseline. PAP was required to be withdrawn for 7 days before each PSG test, at baseline, Week 24 and 52, to limit the impact of PAP on the PSG results at these three endpoints. While differences in PAP adherence between orforglipron and placebo groups could potentially bias estimates of treatment effect on non-PSG outcomes, the collection of adherence data will permit assessment of this potential bias. Moreover, changes in PAP adherence reflect real-world usage patterns, making the results more generalizable.

Overall, despite these limitations, we believe ATTAIN-OSA has the potential to make a meaningful clinical impact by evaluating an oral, scalable therapeutic option that could expand access to effective OSA treatment.

## 5 Conclusion

The ATTAIN-OSA trials are designed to evaluate whether orforglipron, a once-daily oral GLP-1 RA, can provide an effective and accessible treatment option for adults with moderate-to-severe OSA who have overweight or obesity. An oral therapy may offer meaningful advantages over injectable agents, including improved feasibility in under-resourced settings and greater acceptability among individuals who are hesitant about injections or face barriers to injectable treatments [21]. By enabling individuals to choose a formulation that aligns with their preferences and circumstances, orforglipron has the potential to enhance treatment uptake and adherence. These factors create a strong scientific and practical rationale for investigating whether an oral GLP-1 RA such as orforglipron can achieve therapeutic benefits comparable to those demonstrated by injectable incretin-based therapies and thereby expand access to effective OSA management.

## Funding

This study was funded by 10.13039/100004312Eli Lilly and Company.

## CRediT authorship contribution statement

**Atul Malhotra:** Conceptualization, Data curation, Formal analysis, Methodology, Project administration, Supervision, Validation, Visualization, Writing – original draft, Writing – review & editing. **Daniel J. Gottlieb:** Methodology, Supervision, Writing – review & editing. **Vaishnavi Kundel:** Methodology, Supervision, Writing – review & editing. **Holger Woehrle:** Supervision, Validation, Writing – review & editing. **Sujatro Chakladar:** Formal analysis, Methodology, Writing – original draft, Writing – review & editing. **Livia Firmino Goncalves:** Methodology, Project administration, Writing – review & editing. **Giulia Calamai:** Data curation, Project administration, Writing – original draft, Writing – review & editing.

## Declaration of competing interest

The authors declare the following financial interests/personal relationships which may be considered as potential competing interests: Atul Malhotra reports financial support was provided by University of California San Diego (UCSD). Atul Malhotra reports a relationship with Eli Lilly that includes: consulting or advisory. Dr. Malhotra is funded by the NIH, including PI on R01 AG063925, R01 HL148436, R01 HL148436, R01 HL157985, R01 HL166485. He reports income from Livanova, Zoll, Sunrise, Eli Lilly and Company, and Powell Mansfield. He is co-founder and has equity in Clairyon a small startup related to predictive analytics. Resmed provided a philanthropic donation to UCSD in 2015.

Livia Firmino Goncalves reports administrative support, statistical analysis, and writing assistance were provided by Eli Lilly and Company. Livia Firmino Goncalves reports a relationship with Eli Lilly and Company that includes: employment, equity or stocks, and non-financial support.

Daniel J. Gottlieb reports financial support was provided by Eli Lilly and Company. Daniel J. Gottlieb reports a relationship with Signifier Medical Technologies, Inc. that includes: consulting or advisory. Daniel J. Gottlieb reports a relationship with Apnimed Inc that includes: consulting or advisory. Daniel J. Gottlieb reports a relationship with Takeda Development Center Americas Inc that includes: consulting or advisory. Daniel J. Gottlieb reports a relationship with Eli Lilly and Company that includes: consulting or advisory. Daniel J. Gottlieb reports income from Signifier Medical Technologies, Inc., Apnimed, Inc., Takeda Development Center Americas, Inc., and Eli Lilly and Company.

Vaishnavi Kundel reports financial support was provided by Eli Lilly and Company. Vaishnavi Kundel reports a relationship with Apnimed Inc that includes: consulting or advisory. Vaishnavi Kundel reports a relationship with ZOLL Respicardia Inc that includes: consulting or advisory. Vaishnavi Kundel reports a relationship with Eli Lilly and Company that includes: consulting or advisory. Vaishnavi Kundel reports income from Eli Lilly and Company, Apnimed, and ZOLL Respicardia Inc.

Holger Woehrle reports financial support was provided by Eli Lilly and Company. Holger Woehrle reports a relationship with Eli Lilly and Company that includes: consulting or advisory. If there are other authors, they declare that they have no known competing financial interests or personal relationships that could have appeared to influence the work reported in this paper.

Sujatro Chakladar reports financial support was provided by Eli Lilly and Company. Sujatro Chakladar reports a relationship with Eli Lilly and Company that includes: employment and equity or stocks.

Giulia Calamai reports financial support was provided by Eli Lilly and Company. Giulia Calamai reports a relationship with Eli Lilly and Company that includes: employment and equity or stocks.

## Data Availability

Lilly provides access to all individual participant data collected during the trial, after anonymization, with the exception of pharmacokinetic or genetic data. Data are available to request 6 months after the indication studied has been approved in the US and EU and after primary publication acceptance, whichever is later. No expiration date of data requests is currently set once data are made available. Access is provided after a proposal has been approved by an independent review committee identified for this purpose and after receipt of a signed data sharing agreement. Data and documents, including the study protocol, statistical analysis plan, clinical study report, blank or annotated case report forms, will be provided in a secure data sharing environment. For details on submitting a request, see the instructions provided at www.vivli.org.
